# Natural product-based interventions for thyroid disorders: mechanisms and applications

**DOI:** 10.1007/s00210-025-04604-y

**Published:** 2025-10-16

**Authors:** Radwa H. El-Akad, Ahmed H. Elosaily, Noha M. Gamil, Rana M. Merghany, Riham A. El-Shiekh, Wesam Taher Almagharbeh, Hebatollah E. Eitah

**Affiliations:** 1 Pharmacognosy Department, Pharmaceutical and Drug Industries Research Institute, National Research Centre, P.O. Box 12622, Giza, Dokki, Egypt; 2 Department of Pharmacognosy, Faculty of Pharmacy, Ahram Canadian University, Cairo, Egypt; 3 Department of Pharmacology and Toxicology, Faculty of Pharmaceutical Sciences and Drug Manufacturing, Misr University for Science and Technology, 6Th of October City, Egypt; 4 Department of Pharmacognosy, Faculty of Pharmacy, Cairo University, Cairo, 11562 Egypt; 5 Medical and Surgical Nursing Department, Faculty of Nursing, University of Tabuk, Tabuk, 71491 Saudi Arabia; 6 Medicinal and Pharmaceutical Chemistry Department, Pharmacology Group, National Research Centre, Giza, Dokki, Egypt

**Keywords:** Herbal recipes, Thyroid diseases, Malfunctions of thyroid, Endocrine glands

## Abstract

Thyroid diseases are widespread endocrine disorders that affect a significant portion of the global population. The pathology associated with specific types or stages of thyroid disease is complex and intricately linked to various biological functions. While the mortality rate associated with thyroid dysfunction is relatively low, it can lead to metabolic and immunological disorders that result in considerable discomfort for affected individuals. Currently, numerous pharmaceutical options are available for managing thyroid disease; however, issues such as drug toxicity and prolonged treatment durations highlight the urgent need for more effective alternatives. In this review, we conducted a comprehensive literature search to explore the use of herbs and herbal formulations in the treatment of thyroid diseases. Our findings underscore the potential of these natural remedies in drug discovery efforts. It is evident that further scientific investigation into the mechanisms of action of these medicinal plants is crucial for validating their traditional applications. By enhancing our understanding of how these natural products function, we can pave the way for innovative therapeutic strategies that may improve outcomes for individuals suffering from thyroid disorders.

## Introduction

As the largest endocrine gland in the human body, the thyroid plays a crucial role in regulating growth and metabolism by synthesizing thyroid hormones (Zhang et al. 2022a, b). Dysfunction of the thyroid can lead to serious conditions such as goiter, autoimmune thyroid disease (AITD), and thyroid cancer. In recent years, the incidence rates of AITD and thyroid cancer Have risen significantly, reaching approximately 5% and 20%, respectively (Antonelli et al. 2015; Kim et al. 2020, Haddad et al., 2023). Among these disorders, goiter is commonly observed in the general population and can be presented as either diffuse or nodular types based on thyroid histology. The etiology of goiter is complex and can occur in individuals with euthyroid, hyperthyroid, or hypothyroid states (Knobel 2016). While many individuals with goiter lead normal lives, some experience discomfort due to symptoms such as pain or obstruction of the airway and esophagus (Oo). Additionally, nodules may develop in advanced cases of goiter, potentially exacerbating the condition and increasing the risk of thyroid cancer (Yildirim Simsir et al. 2020). Therefore, there is an urgent need for medications aimed at preventing and intervening in the progression of goiter. Treatment options vary based on the type and stage of goiter and may include medications or surgical interventions. Common pharmacological treatments include antithyroid drugs such as propylthiouracil (PTU), methimazole (MMI), carbimazole, and levothyroxine (LT4) (Ling et al. 2019). While surgery provides a rapid means of alleviating symptoms and allows for histological examination of tissues, it is invasive and carries risks such as recurrent laryngeal nerve injury or hypoparathyroidism (Jhajharia 2018). Radioactive iodine therapy serves as a viable alternative to surgery but necessitates extended hospitalization and clinical follow-up post-treatment (Slonimsky and Tulchinsky 2020). Numerous studies have documented the use of herbal extracts for treating thyroid-related diseases globally. The efficacy of both combination therapies and individual herbal treatments has been extensively researched. Results indicate that combination therapies yield significant therapeutic benefits for managing thyroid disorders, suggesting that plant-based medicines may offer multi-target effects with gentler efficacy profiles. Consequently, they are well-suited for integration with conventional Western treatments to provide safer and more effective management of thyroid diseases (Zhang et al. 2022a, b). This review aims to lay the groundwork for further research into the application of herbal formulations in treating thyroid conditions.

## Literature search methodology

A comprehensive Literature search was conducted to identify relevant studies for this review. Electronic databases including PubMed, Web of Science, Scopus, and Google Scholar were systematically searched from inception through June 2024. The search strategy employed a combination of keywords and Medical Subject Headings (MeSH) terms related to thyroid disorders (“hyperthyroidism”, “hypothyroidism”, “Graves’ disease”, “Hashimoto’s thyroiditis”, “goiter”, “thyroid nodules”, “thyroid cancer”) and natural products (“medicinal plants”, “herbal medicine”, “phytochemicals”, “natural products”, “plant extracts”) alongside specific plant names mentioned in the text. The reference lists of retrieved articles were also manually searched to identify additional relevant publications. The search was limited to articles published in English, with a focus on recent studies (2010–2024) while including seminal older works for historical context. Both preclinical (in vitro and in vivo) and clinical studies were considered for inclusion to provide a broad perspective on the current state of evidence. This methodology ensured a thorough and unbiased coverage of the available literature on natural interventions for thyroid disorders.

### Overview of the thyroid gland

The thyroid gland is located in front of the neck and weighs about 15–20 g in an adult person. It is a small butterfly-shaped gland that produces thyroxine (T4) and triiodothyronine (T3) and releases them in the circulation. These hormones affect basal metabolic and linear growth, neural development, brain function and memory, and bone development (Felsenfeld and Levine 2015; Larsen 2015).

Thyroxine (T4) and triiodothyronine (T3) are produced by the thyroid gland. Additionally, calcitonin is a 32 amino acid hormone secreted by the C-cells of the thyroid gland in response to elevated levels of calcium in the serum and gastrointestinal hormone gastrin. It inhibits calcium secretion and protects against hypercalcemia (Mullur et al. 2014).

### Physiological effects mediated by thyroid hormones

Thyroid hormones (T3 and T4) are essential for metabolism. They regulate the metabolic rate, influencing how the body converts food into energy. In addition, they help maintain body temperature by regulating heat in tissues. Moreover, they are critical during childhood for the proper growth and development of bones and tissues (Bassett and Williams 2016), Fig. 1.

**Fig. 1:**
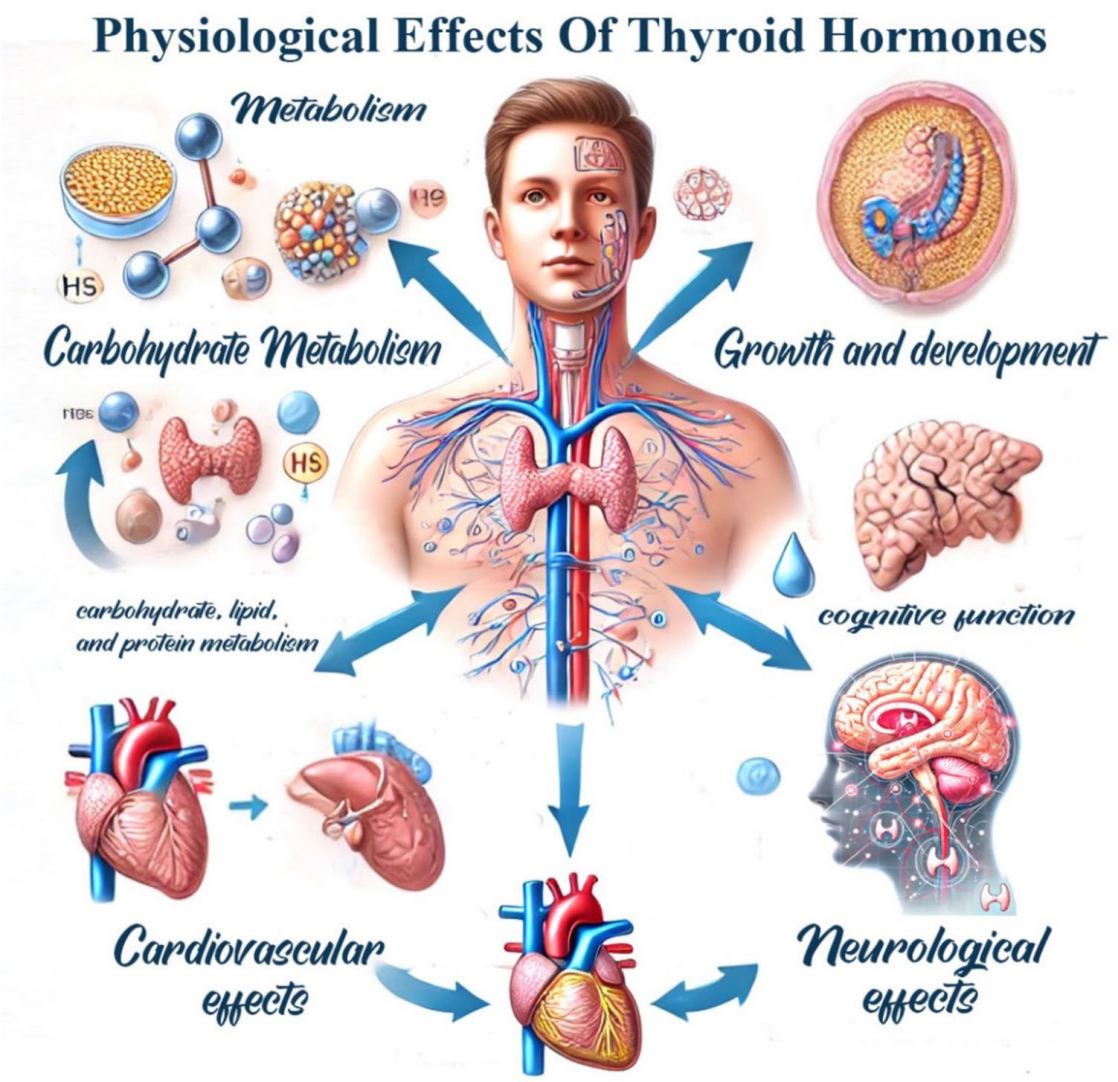
Physiological effects of thyroid hormones

The interaction between thyroid hormones and nuclear receptors influences a wide array of physiological processes:

- Metabolism and digestive system: thyroid hormones increase the basal metabolic rate by enhancing mitochondrial activity and oxygen consumption. They also regulate the synthesis of enzymes involved in oxidative metabolism. The thyroid hormones regulate the body’s metabolism and affect the body weight, which may lead to weight gain in hypothyroidism and weight loss in hyperthyroidism. Additionally, the thyroid gland affects glucose metabolism and insulin sensitivity. Moreover, the proper thyroid function contributes to digestive health. Hypothyroidism can result in constipation, while hyperthyroidism may lead to increased bowel movement frequency (Bassett and Williams 2016).
- Growth and development: in developing tissues, thyroid hormones are essential for proper brain development, skeletal growth, and nervous system maturation (Yau et al. 2019).
- Thermogenesis: T3 regulates the expression of uncoupling proteins (UCPs) in brown adipose tissue, leading to increased heat production. This is especially important for maintaining body temperature in cold environments (Yamakawa et al. 2021).
- Cardiac function: thyroid hormones influence the expression of genes that regulate heart rate and contractility. They increase the expression of beta-adrenergic receptors, leading to enhanced cardiac output. Adequate thyroid hormone levels promote a healthy heart rate and contribute to overall cardiovascular health. Insufficient hormone levels can lead to bradycardia (slow heart rate), while excessive levels can cause tachycardia (rapid heart rate) (Wang et al. 2023).
- Thermogenesis: T3 regulates the expression of uncoupling proteins (UCPs) in brown adipose tissue, leading to increased heat production. This is especially important for maintaining body temperature in cold environments (Yamakawa et al. 2021).

Gene expression modulation is the main mechanism of TH by binding the active hormone T3 to specific nuclear receptors. The spatiotemporal regulation of gene expression involved in Many developmental and physiological processes is a complex process attributed to TH action. These processes include neural cell proliferation and differentiation, cortical lamination, and neuronal migration. The monocarboxylate transporter 8 (MCT8) and the organic anion transporting polypeptide 1C1 (OATP1C1) are the most important transporters for thyroid hormones in the CNS (Mazzilli et al. 2023).

- Reproductive system: thyroid hormones are essential for reproductive health, affecting menstrual cycles and fertility (Condorelli et al. 2019). Pregnancy significantly affects the thyroid gland, and female infertility has been closely linked to thyroid disturbances. Many studies have investigated the TSH reference range in pregnancy, fertility, and assisted reproduction technologies (ART), for the large debate on TSH in these circumstances. Additionally, thyroid hormones were reported to affect spermatogenesis, Leydig, and Sertoli cells. Moreover, the effect of thyroid hormones on the reproductive system in males has been widely debated (Ruder et al. 1971; Fumel et al. 2015; Visser 2018).

### Thyroid hormone biosynthesis and regulation

**Fig. 2:**
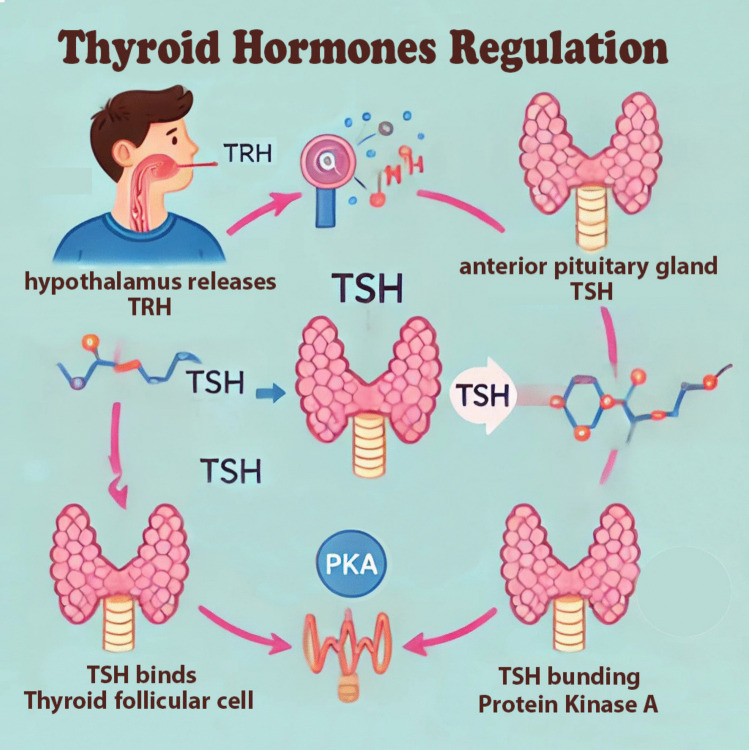
Thyroid hormone regulation

The hypothalamic-pituitary-thyroid axis is a self-regulatory circuit consisting of the thyroid gland, anterior pituitary gland, and hypothalamus. The thyroid gland secretes triiodothyronine (T3) and tetraiodothyronine (thyroxine, T4). The hypothalamus secretes thyrotropin-releasing hormone (TRH), and the anterior pituitary gland releases the thyroid-stimulating hormone (TSH). TRH, TSH, and T4 work synchronously to achieve harmony and maintain proper feedback mechanisms and homeostasis, Fig. 2. A negative feedback mechanism involving the hypothalamus, pituitary gland, and thyroid gland regulates thyroid hormones secretion as follows:

- The hypothalamus secretes TRH (thyrotropin-releasing hormone), which stimulates TSH (thyroid-stimulating hormone) release from the pituitary gland.
- The release of thyroid hormones T3 and T4 from the thyroid gland is stimulated by TSH.
- TRH and TSH release are inhibited by high levels of T3 and T4 which maintains hormone levels within a normal range.

The hypothalamus is the starting point in thyroid hormone regulation; it releases TRH into the hypothalamic-hypophyseal portal system to the anterior pituitary gland. The thyrotropin cells in the anterior pituitary are stimulated to release TSH when TRH is secreted. TRH binds to the anterior pituitary gland TRH receptors, resulting in a G-protein coupled receptor signal cascade. The G-protein coupled receptors activate phosphoinositide-specific phospholipase C (PLC). PLC hydrolyzes phosphatidylinositol 4,5-P(PIP) into 1,2-diacylglycerol (DAG) and inositol 1,4,5-triphosphate (IP). Intracellular calcium stores are mobilized, and protein kinase C is activated by these second messengers, leading to downstream gene activation and TSH transcription. After the release of TSH into the blood, it binds to its receptor (TSH-R) on the basolateral aspect of the thyroid follicular cell. TSH-R stimulation leads to adenylyl cyclase activation and leveling up of the intracellular levels of cAMP which subsequently leads to protein kinase A (PKA) activation. Consequently, different proteins with variable functions are phosphorylated with PKA (Larsen 2015; Núñez et al. 2017).

**Fig. 3:**
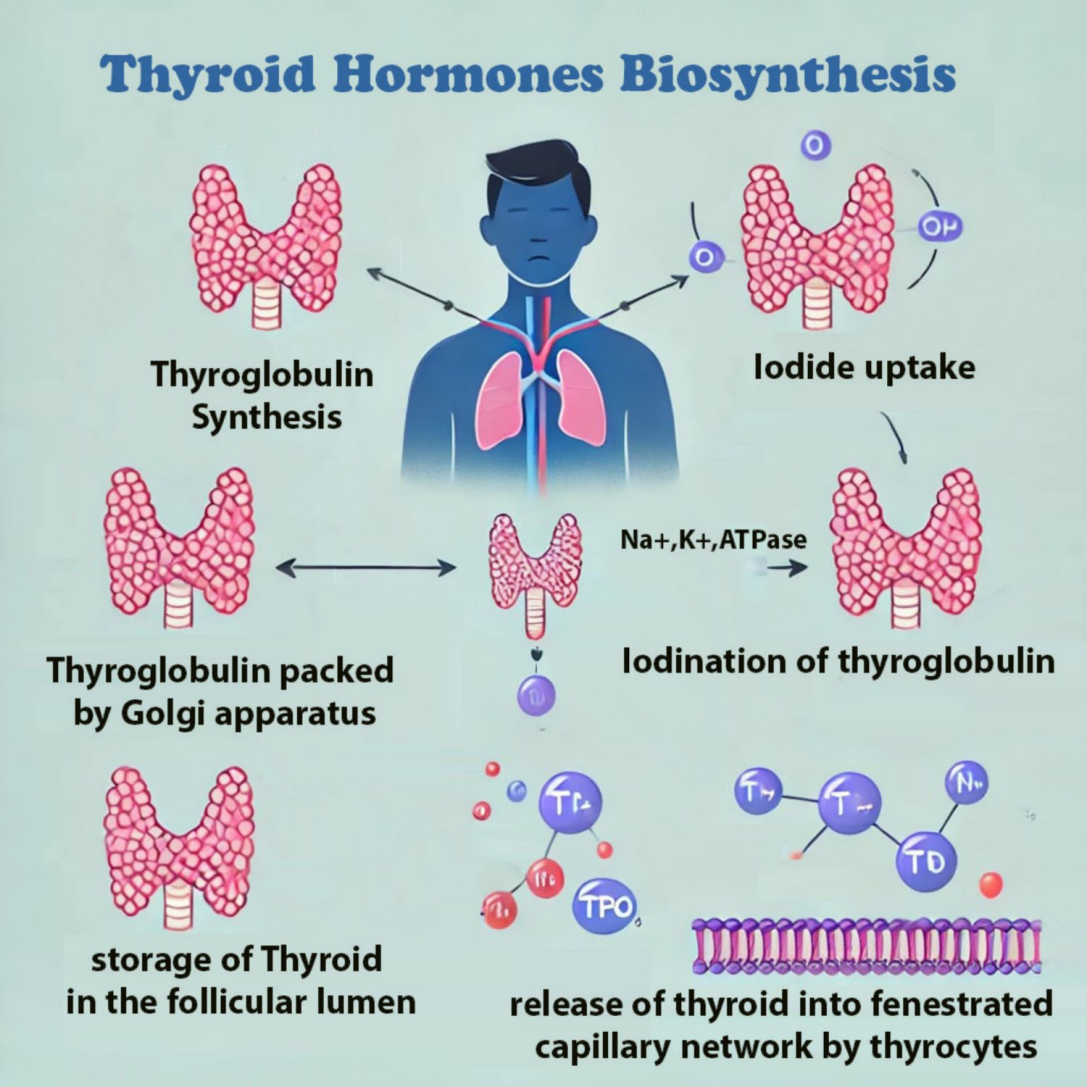
Thyroid hormone biosynthesis

The synthesis of the different thyroid hormones occurs in 5 steps as in Fig. 3.

1. Thyroglobulin synthesis: thyroglobulin (TG) protein is produced by thyroid follicles. The precursor protein TG does not have iodine, and it is produced and packed in the rough endoplasmic reticulum and the Golgi apparatus, respectively, and then stored in the lumen of follicles via exocytosis.
2. Iodide uptake: the activity of Na + -K + -ATPase driven basolateral Na + -I- symporters increases due to Protein kinase A phosphorylation, to transfer iodide to the thyrocytes from the circulation. The iodide diffused to the apex of the cell from the basolateral side, where it is transported into the colloid through the pendrin transporter.
3. Iodination of thyroglobulin: the enzyme thyroid peroxidase (TPO) is phosphorylated and activated by protein kinase. TPO has three functions: oxidation, organification, and coupling reaction.

In oxidation, hydrogen peroxide is used by TPO to oxidize iodide (I-) to iodine (I2). Where hydrogen peroxide is generated by NADPH-oxidase.

In organification, the tyrosine residues of thyroglobulin protein are linked with I2 by TPO. Monoiodotyrosine (MIT) and diiodotyrosine (DIT) are generated, where MIT and DIT Have a tyrosine residue with iodine and 2 molecules of iodine, respectively.

In the coupling reaction, the iodinated tyrosine residues are combined by TPO to make triiodothyronine (T3) and tetraiodothyronine (T4), where MIT and DIT join to form T3, and two DIT molecules form T4.

4 Storage: thyroid hormones are stored in the follicular lumen bound to thyroglobulin.
5 Release: they are released by thyrocytes into the fenestrated capillary network via multiple steps (Schweizer and Köhrle 2013; Braun and Schweizer 2018; Mallya and Ogilvy-Stuart 2018; Tedeschi et al. 2021).

### Cellular mechanisms of thyroid hormones and interaction with nuclear receptors

Thyroxine (T4) and triiodothyronine (T3) play crucial roles in metabolic processes regulation, growth, and development. These hormones bind to and interact with nuclear receptors to finally exert their effect. Most of the time, thyroid hormones are available in the bloodstream bound to plasma proteins such as thyroid-binding globulin (TBG). However, only the free, unbound form of the hormone is biologically active. Once released from the binding proteins, T3 and T4 enter target cells through membrane transporters. T3 is the active form of the thyroid hormone, whereas T4 is converted into T3 in target tissues (such as the liver and kidney) through deiodination by the deiodinase enzymes. Specific transport proteins like MCT8 (monocarboxylate transporter 8) and OATP1C1 (organic anion-transporting polypeptide 1C1) (Zhang et al. 1992).

Intracellularly, T4 is converted to T3 by type 1 or 2 deiodinase enzyme (depending on tissue type). After that, T3 binds to thyroid hormone receptors (TRs, nuclear receptor superfamily). There are two main types of thyroid hormone receptors, TRα and TRβ, which form homodimers or heterodimers with other nuclear receptor family members, the retinoid X receptors (RXRs) (Harvey et al. 2007). Typically, thyroid hormone receptors are in the target cell nucleus, although they can also be found in cytoplasm in an inactive state. The binding of T3 to TRs induces a conformational change in the receptor, which allows the receptor to bind to thyroid hormone response elements (TREs, specific DNA sequences) in the promoter regions of target genes (Simons 2003). Once T3 binds to TRs, it activates or represses the transcription of many genes. Various coactivators or corepressors like CBP/p300 and nuclear receptor corepressor (NCoR), respectively, that modulate gene transcription are recruited by the receptor-ligand complex (Lee et al. 1995; Luca et al. 2003). In the presence of T3, TRs interact with coactivators like TRAP (thyroid receptor-associated protein), which increases gene transcription; meanwhile, in the absence of T3, corepressors that inhibit gene transcription are recruited by TRs. The changes in gene transcription led to changes in the synthesis of proteins that affect various physiological processes, including metabolism, development, and growth. These include proteins involved in oxygen consumption, mitochondrial function, thermogenesis, and lipid and carbohydrate metabolism (Sorisky 2016).

### Common thyroid diseases

#### Hypothyroidism

Hypothyroidism is a decreased thyroid gland activity typically manifested as cold intolerance, bradycardia, constipation, weight gain, and fatigue. Decreased iodine intake can cause iodine deficiency and decreased thyroid hormone synthesis. Iodine deficiency can cause goiter, cretinism, myxedema coma, and hypothyroidism, Table 1 (Sharma et al. 2021, Khan et al. 2023). Hypothyroidism can cause slowed speech, impaired memory, and sleepiness. Hypothyroidism Has 3 types: primary (caused by the thyroid gland and induces a compensatory TSH increase), secondary (caused by pituitary disorders and results in decreased release of TSH and T3/T4 levels), and tertiary hypothyroidism (caused by hypothalamic disorders), resulting in decreased levels of TRH, TSH, and T3/T4 (Shahid et al. 2024).

**Table 1:** Different levels of TSH, T3, and T4, common symptoms, and prevalence in different thyroid disorders

Disorder	TSH levels	T3 levels	T4 levels	Common symptoms	Prevalence
Hypothyroidism	High	Low	High	Fatigue, weight gain, cold intolerance	Common, especially in women
Hyperthyroidism	Low	High	High	Weight loss, anxiety, heat	Less common, may occur in women and men
Hashimoto’s thyroiditis	High (variable)	Normal/low	Normal/low	Fatigue, sensitivity to cold, goiter	Common, autoimmune condition
Graves’ disease	Low	High	High	Weight loss, increased appetite, anxiety	Less common, often in women

#### Hyperthyroidism

Hyperthyroidism is caused by increased thyroid gland activity manifested as heat intolerance, weight loss, diarrhea, muscle weakness, and fine tremors. Hyperthyroidism can lead to irritability and hyperexcitability, Table 1. Free T4 levels are abnormally increased, and TSH levels are decreased in primary hyperthyroidism. Thyroid gland disorders can result in excess production of T3 and T4 with compensatory TSH reduction. In addition, the unregulated TSH secreted in thyrotroph adenoma can lead to increased production of T3 and T4. Moreover, in some conditions there may be an ectopic production of thyroid hormones, leading to increased thyroid hormones and a compensatory decrease of TSH, Table 1 (Oo KM n.d.; Wiersinga et al. 2023; Shahid et al. 2024).

#### Autoimmune thyroid disorders

- Hashimoto’s thyroiditis

Hashimoto thyroiditis is the most common cause of hypothyroidism in iodine-sufficient areas, and it is caused by thyroid gland destruction due to autoimmune disturbance, where the thyroid follicular cells are subjected to death by the effect of CD8 + T cells. TH1 cells release IFN-gamma which recruits and activates macrophages. Hashimoto’s thyroiditis patients may develop a symmetrical, non-tender, and painless goiter during the early stage of the disease. Thyroid follicles are damaged and may be ruptured during inflammation. If rupture of the thyroid follicles occurs, the patient may experience Hashitoxicosis (thyroid hormone from ruptured follicles, causing symptoms of hyperthyroidism) or be asymptomatic. During the progression of the disease, the size of the thyroid gland May decrease or remain normal, according to fibrosis extent. This may result in hypothyroidism symptoms. Anti-thyroid autoantibodies are produced in addition to cell-mediated destruction, which results in antibody-dependent cell-mediated cytotoxicity. Diagnosis of Hashimoto thyroiditis is done by ultrasound, thyroid function testing, and antibody detection. To exclude Malignancy, fine-needle aspiration and radioactive iodine uptake tests can be performed 26, (Ragusa et al. 2019).

- Graves’ disease

The most common cause of hyperthyroidism is Graves’ disease. It is an autoimmune disease caused by the production of antibodies against TSH receptors, which leads to increased thyroid function and growth. In this disease, patients will have abnormally high levels of T4 and T4 and decreased TSH. The diagnosis is confirmed by a positive test for the TSH-receptor IgG immunoglobulin. Most times, symptoms of diffuse goiter and hyperthyroidism are present. Orbital fibroblasts may be activated by these antibodies, leading to proliferation of fibroblasts and differentiation to adipocytes. Consequently, hyaluronic acid and glycosaminoglycan (GAG) production increases leading to an increased volume of Muscle tissue and intraorbital fat. It causes Lid retraction, exophthalmos, and diplopia Due to ocular motility problems. Additionally, pretibial myxedema occurs Due to dermal fibroblast stimulation that leads to GAGs deposition in the connective tissue. 70% of patients with Graves’ disease have elevated anti-TPO antibodies (Lanzolla et al. 2024, Shahid et al. 2024).

### Prevalence and stats for thyroid problems

**Fig. 4:**
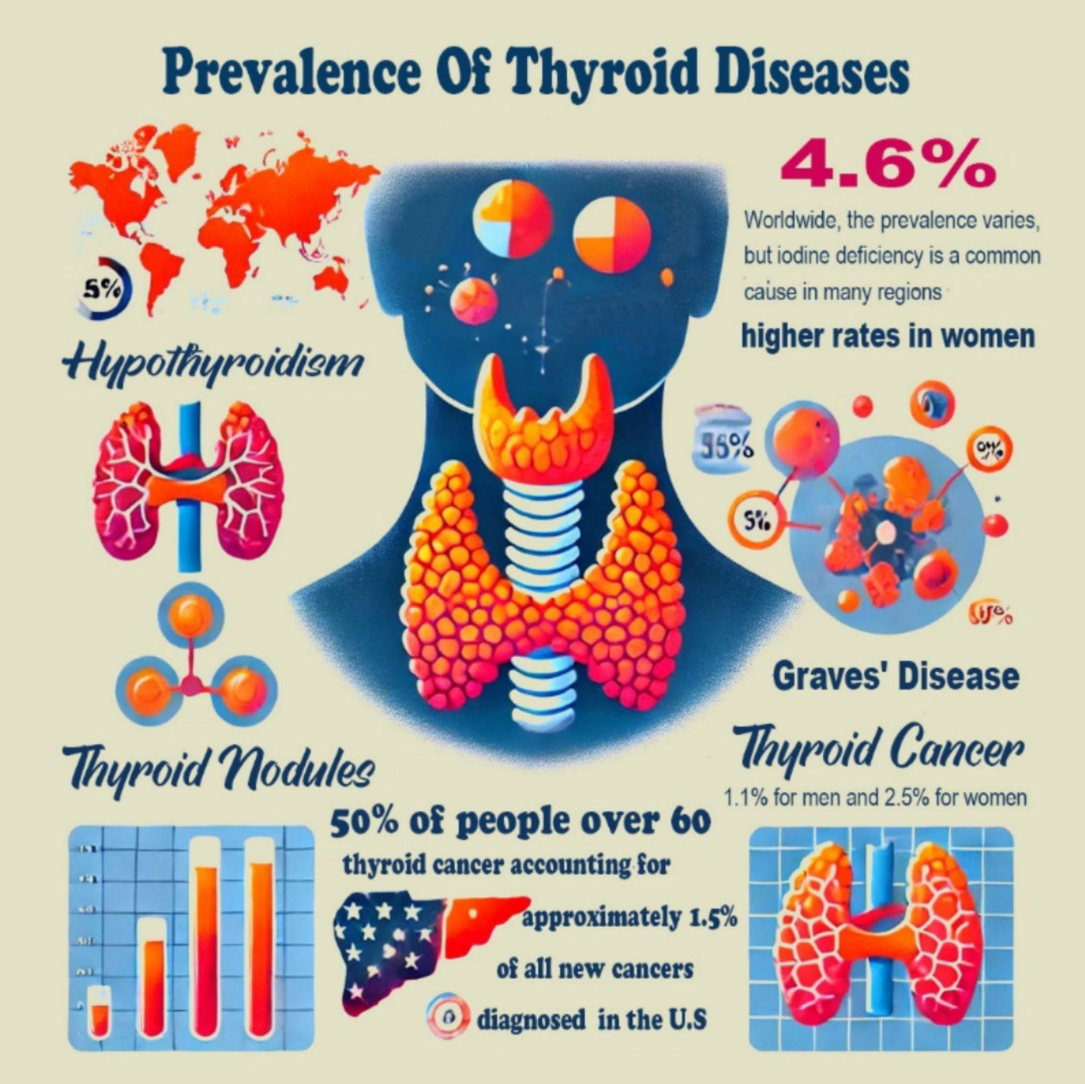
Prevalence and statistics of thyroid diseases

The prevalence of thyroid diseases varies depending on many factors including age, gender, and geographical area, Fig. 4.

- Hypothyroidism: worldwide, the prevalence varies, but iodine deficiency is a common cause in Many regions. It affects approximately 4.6% of the US population, with higher rates in women (about 10% in women over 60 years) (Wyne et al. 2023).
- Hyperthyroidism: affects about 1% of the US population, with a higher prevalence in women (Doubleday and Sippel 2020). Graves’ disease accounts for up to 80% of hyperthyroidism cases (Sharma 2022).
- Goiter: the prevalence of goiter can vary widely depending on iodine intake. In areas with iodine deficiency, the prevalence can be as high as 10–20% (Hatch-McChesney and Lieberman 2022).
- Thyroid nodules: thyroid nodules are common and May be found in about 50% of people over 60 when assessed with ultrasound, but only a small percentage (5–15%) are cancerous (Desser and Kamaya 2009).
- Thyroid cancer: incidence is increasing, with thyroid cancer accounting for approximately 1.5% of all new cancers diagnosed in the USA each year. The Lifetime risk of developing thyroid cancer is about 1.1% for Men and 2.5% for women (Pellegriti et al. 2013).

### Risk factors, diagnosis, and complications

#### Risk factors for thyroid problems

1. Gender: women are significantly more likely to experience thyroid problems than Men, with conditions Like hypothyroidism occurring roughly 5–8 times more frequently in women (Stoll 2019; Abdulla et al. 2020).
2. Age: the risk of developing thyroid disorders increases with age, particularly for hypothyroidism (Taylor et al. 2023).
3. Autoimmune thyroid disease represents a risk of Hashimoto’s thyroiditis and Graves’ disease (Franco et al. 2013).
  Hashimoto’s thyroiditis: the most common cause of hypothyroidism, seen in about 1–2% of the US population.
  Graves ‘disease: affects about 0.5–3% of the population.
4. Family history: a history of thyroid disease in the family can increase risk (Memon et al. 2004).

#### Diagnosis of thyroid diseases

Thyroid disorder diagnosis is achieved via multiple steps: clinical evaluation, laboratory tests, and imaging studies (Demers 2004, Pappa T 2018).

1. Clinical evaluation:
  Patient history: symptoms such as weight changes, energy levels, temperature sensitivity, and emotional well-being can provide clues.
  Physical examination: checking for signs like goiter (enlargement of the thyroid), changes in skin texture, pulse rate, and deep tendon reflexes.

2. Laboratory tests:
  Thyroid function tests.
  TSH (thyroid-stimulating hormone): elevated in hypothyroidism and low in hyperthyroidism.
  Free T4 and T3: free T4 is typically low in hypothyroidism and elevated in hyperthyroidism.
  Antibody tests: anti-thyroid peroxidase (anti-TPO): often elevated in autoimmune thyroid conditions like Hashimoto'’ thyroiditis.
  TSH receptor antibodies: elevated in Graves’ disease.

3. Imaging studies and uptake: measures how much tracer is taken up by the thyroid.
  Ultrasound: is used to assess thyroid nodules, inflammation, or enlargement of the thyroid.
  CT or MRI: may be used if there are concerns about the extension of mass.
  Fine needle aspiration (FNA) biopsy: this is performed when suspicious thyroid nodules to rule out malignancy.

#### Complications of thyroid diseases

1. Hypothyroidism complications:
  Myxedema coma: a severe, life-threatening form of hypothyroidism with symptoms such as extreme cold intolerance, drowsiness, and mental impairment. It occurs due to persistent hypothyroidism and loss of adaptive mechanisms to restore homeostasis (Elshimy et al. 2019).
  Cardiovascular issues: increased risk of heart disease due to high cholesterol levels (Zúñiga et al. 2024).
  Infertility and birth defects: these can affect menstrual cycles and lead to complications in pregnancy (Tudosa et al. 2010).
2. Hyperthyroidism complications:
  Thyroid crisis (thyroid storm): a rare but life-threatening condition characterized by extremely high levels of thyroid hormones, leading to fever, tachycardia, and confusion (Singh 2024).
  Osteoporosis: increased thyroid hormone levels can lead to decreased bone density (Delitala et al. 2020).
  Eye problems: Graves’ disease can cause Graves’ ophthalmopathy, leading to bulging eyes, vision problems, or even vision loss (Lanzolla et al. 2024, Shahid et al. 2024).
3. Goiter complications: large goiters can compress the trachea or esophagus, leading to breathing or swallowing difficulties (Brinch et al. 2019).
4. Thyroid nodules:
  Malignancy: although most thyroid nodules are benign, a small percentage can be cancerous. Regular monitoring or intervention is required (Bomeli et al. 2010).
  Hyperthyroidism: some nodules can cause excess thyroid hormone production, leading to hyperthyroidism
5. Mental health issues:

Both hypothyroidism and hyperthyroidism can contribute to anxiety, depression, or mood swings (Nuguru et al. 2022).

### Animal model, induction method, mechanisms of action, and main targets of each disorder

The use of animals in scientific research has long been a cornerstone of biological and medical studies, though it remains a subject of significant ethical debate. The close anatomical and physiological similarities between humans and other mammals have made animals invaluable models for investigating a wide range of biological processes and testing new treatments before they are applied to humans (Mukherjee et al. 2022). Animal models have been crucial in advancing our understanding of various diseases and conditions, from basic biological mechanisms to the development of vaccines and novel therapies. This is especially evident in conditions that affect both humans and animals (Kiani et al. 2022). The similarities in disease mechanisms across species are striking, and as a result, many veterinary drugs used to treat animals are either identical or closely related to those used for human treatment. This shared biological foundation underscores the importance of animal research in driving medical advancements and improving public health (Domínguez-Oliva et al. 2023).

Hyperthyroidism: animal models of hyperthyroidism are the most used in the study of pathophysiology and therapeutic possibilities of this condition, characterized by overproduction of thyroid hormones. Rodent models, generally rats and mice, are the most common; their hyperthyroid state may be induced by supplementation of thyroid hormones such as thyroxine (T4) or triiodothyronine (T3), or by the administration of thyroid-stimulating immunoglobulins to induce Graves’ disease, one of the autoimmune forms of hyperthyroidism (Zhang et al. 2022a, b). These models have an increased metabolic rate, tachycardia, and weight loss due to overstimulation of the thyroid gland. Such symptoms have resulted from the over-stimulation of the thyroid due to the induction of hyperthyroidism through exogenous thyroid hormones and in turn cause excessive secretion of thyroid hormones, which disturb the feedback loop of the hypothalamic-pituitary-thyroid axis (Bao et al. 2023). These perceptions about the changes in receptors, thyroid hormone metabolism, and synthesis have been augmented by research involving transgenic mouse models that have thyroid hormone receptors and TSH receptor mutations. The main mechanism of development of hyperthyroidism, according to Eckstein et al. (2020) (Eckstein et al. 2020), in these models was the overstimulation of thyroid follicular cells due to either the presence of TSIs or excess thyroid hormone, which leads to an increase in thyroid hormone secretion. These models allow researchers to zero in on and study the target TSH receptor, thyroid follicular cells, and the feedback mechanisms along the HPT axis to gain insight into how dysregulated thyroid hormone signaling contributes to metabolic disturbances associated with hyperthyroidism Kohn et al. (2019) (Kohn et al. 2019).

Hypothyroidism: hypothyroidism is a condition in which thyroid hormones are produced in insufficient amounts, and it is one of the most common animal models for studying its mechanisms and assessing therapeutic interventions. The induction of hypothyroidism commonly involves the use of propylthiouracil (PTU), an inhibitor of thyroid hormone synthesis, iodine deficiency, or thyroidectomy (Głombik et al. 2021). Congenital hypothyroidism is also caused by genetic manipulations, including mutations in TR genes in the rodent models (Głombik et al. 2022). Such models are useful in studying metabolic and developmental consequences of thyroid hormone deficiencies, giving insight into how thyroid hormone insufficiency impacts growth and neurological function. Mechanisms include a decline in the level of thyroid hormones that promotes compensatory increases in TSH secretion from the pituitary gland. The resulting elevated TSH promotes thyroid gland enlargement, or goiter, with metabolic slowing in affected animals. Targeted molecular pathways involved include thyroid follicular cells, TSH receptors, and thyroid hormone receptors. Animal models of developmental thyroid hormone deficiency have also reproduced marked neurocognitive impairment and growth retardation in offspring, much like in humans with hypothyroidism (Chaulin et al. 2021). These models permit the testing not only of thyroid hormone replacement but also act as a vehicle to test putative interventions for developmental deficits due to thyroid hormone insufficiency (Kim et al. 2024).

Thyroid nodules: thyroid nodules can either be benign or malignant; the latter are subjected to study in animal models for the elucidation of mechanisms leading to development and progression. Animal models in thyroid nodules usually arise from induced genetic mutations or an exposure to substances that may trigger abnormal growth of thyroid cells (Morré et al. 2018). Thyroid hyperplasia and nodular development can be induced by the use of rodent models and transgenic mice with mutations in genes such as Ras or TSH receptor mutations. In particular, Ras mutations induce dysregulated cell division within thyroid follicular cells, leading to hyperplastic thyroid lesions including nodules (Peterson 2014). The second generally used approach is the chronic exposition to high levels of iodine or TSH receptor agonists. This results in thyroid hyperplasia, leading to the formation of nodules (Peterson et al. 2018). The key mechanism of nodule formation is realized through the Ras/MAPK signaling pathway activation, leading to uncontrollable cell proliferation. Besides, PI3K-AKT also plays a role in tumor promotion of thyroid tumors (Darras, Van Herck et al. 2011). These models allow the investigation of molecular pathways underlying tumorigenesis and cellular processes driving nodule formation, providing critical insights into the pathophysiology of thyroid cancer. Indeed, such targeted therapeutic interventions against these signaling pathways may prevent or treat malignant thyroid nodules and associated cancers (Vagney et al. 2018).

Goiter: the pathophysiology of many conditions and the mechanisms of gland dysfunction, such as the abnormal enlargement of the thyroid gland called goiter, can be induced experimentally in animal models. The most common method of inducing goiter in animals is through deficiency in iodine, since iodine is a critical element in thyroid hormone synthesis (Colque-Caro et al. 2023). These iodine deficiency diets are usually applied in rodent models in an attempt to model human conditions of impaired production of thyroid hormones with subsequent enlargement of the thyroid gland. This is so because, due to a deficiency of iodine, the increased production of TSH stimulates compensatory growth of the thyroid follicular cells for the deficiency of these hormones, resulting in goiter (Kolesnik et al. 2014). Besides, goiter may be experimentally induced in animals exposed to goitrogenic substances, like perchlorate or thiocyanates, which block iodine uptake in the thyroid gland, causing a similar compensatory rise in secretion of TSH and hyperplasia of thyroid cells (Nourani and Sadr 2023). In those models, disturbed iodine availability impairs the synthesis of thyroid hormones, resulting in compensatory enlargement of the thyroid. In all these models, the iodine transporter, thyroid follicular cells, and TSH receptors are the main targets. All these models have provided good insights into iodine deficiency in goiter development and thyroid dysfunction and are critical to an understanding of the global public health problem of iodine deficiency-related thyroid disorders (Kujur et al. 2021).

Hashimoto’s thyroiditis: Hashimoto’s thyroiditis is an autoimmune disease in which the immune system attacks the thyroid gland, leading to hypothyroidism. It is usually induced in animal models by immunization with antigens from the thyroid, such as thyroglobulin or thyroid peroxidase, which induce the production of autoantibodies that attack thyroid cells (Liu et al. 2024). Transgenic mice that develop thyroid-specific autoantigens in a spontaneous manner have, therefore, been very useful and provide a good platform for studying immunological mechanisms of the disease as reported by Merrill and Pandiyan (2018) (Merrill and Pandiyan 2018). The destruction of thyroid follicular cells in Hashimoto’s thyroiditis is mediated via T cells along with concurrent production of autoantibodies against thyroid-specific proteins including TPO and thyroglobulin. The main targets in these models are thyroid follicular cells, immune cells (particularly T lymphocytes), and the autoantibodies that bind to thyroid antigens. These models have provided crucial insights into the immunological pathways that drive autoimmune thyroid diseases and have been essential for developing potential immunomodulatory therapies aimed at modulating the autoimmune response in Hashimoto’s thyroiditis (Qian et al. 2023). Such models will further contribute to the investigation of genetic and environmental factors in the autoimmune development of thyroid disorders and, as such, will be an important tool for therapeutic development (Capuzzo 2021).

Graves’ disease: Graves’ disease is another autoimmune condition of the thyroid characterized by the production of TSIs that acts on TSH receptors found on the plasma membrane of thyroid follicular cells, triggering an excessive response by inducing a surge in secretion that eventually causes hyperthyroidism (Bartalena et al. 2022). Most of the time, Graves’ disease models are done with active immunization using antigens or using transgenic mice to produce TSIs (Davies et al. 2020). These models reproduce quite well in the autoimmune process of Graves’ disease. The Graves’ IgGs or TSIs bind to the TSH receptor and cause the permanent stimulation of thyroid glands that result in the overproduction of thyroid hormones. The autoantibodies in this process against the TSH receptor continuously stimulate the thyroid gland toward hyperplasia and production of increased amounts of hormones (Antonelli et al. 2020). Among the major targets in these models, TSH receptors on thyroid follicular cells take a leading place, while components of the immune system, majorly B and T lymphocytes, produce TSIs. These studies on the models have elucidated how this immune-mediated activation of the thyroid gland leads to systemic effects, such as the presence of goiter or the clinical symptoms of hyperthyroidism, which are weight loss, increased heart rate, and nervousness (Wémeau et al. 2018). These models have provided a service in the development of therapies for Graves’ disease, including immune-modulatory treatments targeting autoimmune components of the disease, and thus laid the groundwork for therapeutic advances in human patients (Kahaly 2020). Summary of different animal models are listed in Table 2.

**Table 2:** Significance and challenges of different animal models in thyroid disorders

Disease model	Animal model	Significance	Challenges	References
**Hypothyroidism**	Rodents (mice, rats)	Models for thyroidectomy, iodine deficiency, and drug testing	Metabolic differences; short lifespan limits chronic studies. Limited complexity of disease	(Rochau et al. 2020)
	Primates (monkeys)	Closely mirrors human thyroid function	High cost, long gestation, species variability in thyroid function	(Schaffner et al. 2021)
	Zebrafish	Good for chemical screening, transparent embryos	Limited lifespan, different thyroid regulation, no full HPT axis	(Chopra et al. 2019)
	Sheep	Similar to human thyroid physiology	High cost, ethical concerns, difficulty replicating iodine deficiency	(Mostaghni et al. 2008)
**Hyperthyroidism**	Rodents (mice, rats)	Models for Graves’ disease, thyroid hormone effects	Genetic models do not fully replicate human disease. Short lifespan limits long-term effects study	(Engels et al. 2016)
	Primates (monkeys)	Similar human hyperthyroidism	Cost, difficult environmental control, small sample size	(Zhu et al. 2024)
**Iodine deficiency**	Rodents (mice, rats)	Common for iodine deficiency studies	Different metabolism, short-term focus, species differences in thyroid adaptation	(Maier, et al. 2007)
	Sheep	Useful for studying iodine deficiency	High cost, difficulty replicating human deficiency levels	(Maier, et al. 2007)
**Thyroid cancer**	Rodents (mice, rats)	Models for thyroid cancer mutations (e.g., BRAF)	Slow tumor progression, genetic limitations, and tumor microenvironment differences	(Matsuu-Matsuyama et al. 2021)
	Primates (monkeys)	Closer to human cancer progression	High cost, difficult to replicate metastasis	(Zhu et al. 2024)
	Pigs (miniature pigs)	Better representation of cancer dynamics	High cost, ethics, difficulty standardizing models	(Shen et al. 2016)
**Autoimmune thyroid disease**	Rodents (mice, rats)	- Models for autoimmune thyroiditis (e.g., NOD mice)	Immunological differences, limited model longevity	(Ma et al. 2021)
	Primates (monkeys)	More accurate for autoimmune thyroid disease	High cost, ethics, difficult to develop naturally occurring disease	(Wang et al. 2013)

The importance of rat and mouse models has proved their outstanding importance in biomedical research. Besides, other mammalian and non-mammalian small domestic animals like the guinea pig, hamster, rabbit, ferrets, birds, amphibians, fishes, flies, and worms have equal importance in terms of anatomical and physiological resemblance with humans. Large animal models also proved their uniqueness due to specific anatomical and physiological characteristics pertinent to those specific researches (Liao et al. 2022).

### Current and emerging treatment approaches and their associated side effects

Treatment options for different thyroid disorders generally depend on disease activity and severity,and it could be accomplished by following different treatment options following the cause of thyroid disorder. For active, moderate or severe disease, a variety of treatment strategies are used, sometimes resulting in short-term relief where advances in both surgical and medical management are continuously evolving (Douglas 2019). For example, the following options may be used: antithyroid drugs (methimazole and propylthiouracil), radioactive iodine, beta-blockers, and surgery (Wiersinga et al. 2023).

#### The major treatment options for different thyroid disorders may include

Hyperthyroidism is generally treated with antithyroid medications, radioactive iodine (RAI) therapy, and, in some cases, surgery. The two most commonly prescribed drugs, methimazole and PTU, reduce the production of thyroid hormones. Although effective, these medications often present such side effects as skin rashes, fever, arthralgia, liver toxicity, and agranulocytosis—an extreme decline in white blood cell count-that preclude their long-term use in certain patients (Mishra et al. 2020). Other alternative treatments include radioactive iodine therapy, which selectively destroys the overactive thyroid tissue, thus leaving many patients in permanent hypothyroidism and requiring lifelong thyroid hormone replacement therapy. While usually effective, RAI is also associated with neck pain, salivary gland inflammation, and a slightly increased risk of radiation-induced malignancy. If medical therapies fail, surgical thyroidectomy may be undertaken but not without its risks: nerve damage, hypoparathyroidism, bleeding, and infection. Although these may be potential side effects, the success rate concerning these treatments is very high, and most patients will achieve symptom relief and normalized thyroid hormone levels (Scarabosio et al. 2024).

Thyroid nodules and goiters are mostly treated according to their size, symptoms, and malignant potential. In cases of benign nodules, a fine needle aspiration (FNA) is usually conducted to assess the nature of the nodule, while RFA or ethanol injections are performed to shrink benign nodules or for symptomatic improvement. Levothyroxine therapy may be given with the hope of suppressing benign nodule growth, though its use is still controversial (Tanase et al. 2020). Long-term use may induce side effects such as cardiac arrhythmias or bone loss. Large and symptomatic nodules or goiters can be treated with surgery when the more conservative approaches have proven fruitless. Complications of surgery include hoarseness, injury to the recurrent laryngeal nerve, hypocalcemia due to damage to the parathyroid glands, and scarring (Genere and Stan 2019). In the case of malignant nodules, thyroidectomy is performed, followed by the administration of radioactive iodine to destroy any residual thyroid tissue, which may induce permanent hypothyroidism. The most common cause of hypothyroidism, Hashimoto’s thyroiditis, is treated with levothyroxine (Burch et al. 2022). However, because it is an autoimmune disease, extra treatments may be necessary, too, such as corticosteroids or immunosuppressants in serious instances, to handle inflammation and autoimmunity. In cases of Graves’ disease, treatment can include antithyroid medications such as methimazole or PTU, which impede thyroid hormone synthesis (Brandfon et al. 2023). Of the two, methimazole is generally preferred because it has fewer side effects than PTU, which can cause liver toxicity in a few patients. When medications are either not effective or poorly tolerated, RAI is employed, which commonly renders the patient hypothyroid, thus requiring lifelong supplementation of thyroid hormones. Surgical resection of part or all of the thyroid is indicated in selected patients but may have serious risks, including nerve damage, infection, and bleeding. Side effects of all these treatments are significant to necessitate individualized care, close monitoring, and a multidimensional approach to the treatment of thyroid disorders (Raza et al. 2021).

Hypothyroidism is usually treated using levothyroxine, a synthetic form of the thyroid hormone thyroxine (T4), to replace the deficient thyroid hormones. This therapy is highly effective in normalizing thyroid hormone levels, relieving symptoms, and improving quality of life. However, the side effects of levothyroxine can occur if the dosage is too high or too low (Patel et al. 2019). Over-replacement is associated with symptoms of hyperthyroidism, including palpitations, weight loss, heat intolerance, and osteoporosis from long-term high doses. Under-treatment may be characterized by persistence of symptoms such as fatigue, depression, cold intolerance, dry skin, and constipation (Wang et al. 2019). Patients require regular monitoring of thyroid function tests to ensure the correct dosage, and adjustment may be needed over time due to factors such as aging, pregnancy, and changes in other health conditions. In rare cases, patients may also experience allergic reactions to levothyroxine or other components of the medication. For the group of hypothyroid patients with precipitating causes, like autoimmune disease, immunosuppressive treatment, or handling of the autoimmune response might also be implemented along with thyroid hormone replacement (Men et al. 2021).

#### Gut microbiota and its impact on thyroid disorders

The gut microbiota (GM) composition is heavily shaped by environmental factors and personal lifestyle choices, in addition to genetic predisposition (Suzuki et al. 2019). It typically remains stable throughout adulthood due to its inherent resilience, capable of returning to its original state after minor disruptions. However, when exposed to long-term disturbances, GM composition can adapt, although substantial perturbations may result in a dysbiotic state (Korach-Rechtman et al. 2019). Though the concept of a “healthy” microbiota is challenging to define, dysbiosis can usually be defined as a certain imbalance where the pathogenic species are dominant over beneficial symbionts and commensals (Hou et al. 2022). Several systemic and organ-specific diseases have been linked with dysbiosis; among those, stronger evidence exists for metabolic disorders, allergies, autoimmune diseases, disturbances of the central nervous system, and some forms of cancer (Fitzgibbon and Mills 2020).

This might be a potential connection to GM and thyroid regulation suggested initially by the Harries (1923) more than hundred years back (Harries 1923). The research regarding it was not very substantial from a long time as being unable to culture or specifically identify the different strains showed hindrance in such scientific research. The discovery of sequencing technologies and improved methods of analysis have seen research into the role of microbiota in thyroid disorders rapidly develop over recent years. Indeed, many original research articles, narrative reviews, and systematic reviews have discussed the composition of GM in patients with different thyroid disorders. Some studies also looked into the interaction between gut microbiota and micronutrients involved in thyroid function and the role of prebiotics-probiotics in modulating microbiota on thyroid function (Gianchecchi and Fierabracci 2019).

However, evidence regarding the role of probiotics and synbiotics is still too scant to recommend their routine use in thyroid disorders (Shao et al. 2023). Moreover, even if certain elimination diets are not recommended, patients should undergo evaluation for potential deficiencies of micronutrients and vitamins, especially in cases when gastrointestinal autoimmune comorbidities feature (Shaheen et al. 2022).

### Limitations and challenges in thyroid disorder management

Thyroid disorders, including hyperthyroidism, hypothyroidism, thyroid nodules, goiter, Hashimoto’s thyroiditis, and Graves’ disease, are subject to several limitations and challenges that pose a barrier to effective diagnosis and treatment and long-term management. A major challenge relates to the heterogeneity of clinical presentation, making it difficult to recognize thyroid disorders at their inception (Mallhi et al. 2023). The most common presentations of thyroid dysfunction include nonspecific complaints such as tiredness, weight change without obvious cause, mood alteration, and gastrointestinal disorders. The symptoms have a very broad differential diagnosis and therefore most cases are being missed or diagnosed lately. Mild forms of thyroid diseases in some patients may be even asymptomatic; therefore, identification of subclinical conditions is going to be more difficult (Korevaar et al. 2017). Due to this fact, thyroid disorders are mostly diagnosed when the diseases have reached a rather worse stage. In particular, autoimmune thyroid diseases, such as Graves’ disease and Hashimoto’s thyroiditis, present a difficult situation since their symptoms can be mistaken for other autoimmune conditions or can even be mistaken for stress-related health problems. This therefore increases the chances of misdiagnosis, delayed appropriate treatment, and poor clinical outcomes (De Sanctis et al. 2019).

In addition, the diagnostic strategy relating to thyroid diseases is also burdened with many limitations due to the lack of uniform protocols relevant to the management of thyroid nodule and goiter. Whereas ultrasound and FNA have been the two most commonly used imaging techniques for thyroid nodule assessment, each has some drawbacks (Sturgeon and Kim 2021). While FNA is a standard diagnostic modality, it does have a recognized risk of false-negative or positive results and cannot always differentiate with certainty benign from malignant nodules (Sawka et al. 2021). In addition, radioactive iodine uptake tests, one of the common ways of assessing thyroid activity, are not always diagnostic, especially in case presentations that are atypical. The fact that benign thyroid nodules are highly prevalent in the general population provides one challenge, in and of itself, to judgment regarding when surgical intervention or other invasive treatments, such as thyroidectomy, is required (Castillo-Gonzalez et al. 2020). Equally, there is now increased recognition of the part that molecular and genetic factors play in the etiology of thyroid cancers, but one of the key research gaps continues to be a lack of reliable biomarkers for distinguishing benign from malignant thyroid lesions. Thus, it is further important to do further studies on new molecular markers and non-invasive diagnostic tests for an improved accurate diagnosis of thyroid cancer (Jaiswal and Gurudiwan 2023).

An important disadvantage concerning treatment is the side effects burden associated with current treatments. It represents one of the critical limiting aspects for current therapies in the management of hyper- and hypothyroidism. The side effects of methimazole or propylthiouracil medication prescribed in hyperthyroidism might also be related to liver toxicity, agranulocytosis, skin rash, or gastrointestinal disturbances; as such, it may notably interfere with patients’ treatment adherence. Moreover, in many cases, the administration of these drugs over a long period is not very effective, and it often leads to the recurrence of the disease (Melo et al. 2022). The RAI therapy, while effective, often causes hypothyroidism, and this requires lifelong replacement with thyroid hormones. Dosage adjustment of thyroid hormone replacements is still an art since inappropriate dosing can result in symptoms of overtreatment or undertreatment with further complications including cardiovascular problems, loss of bone density, and neurological disturbances. Surgical removal of thyroid tissue, thyroidectomy, is lifesaving in many cases but runs risks such as nerve damage, parathyroid dysfunction, and infection. These complications, therefore, result in a dilemma in the treatment modality among clinicians, especially in elderly patients or in the presence of associated co-morbidities (Salmani et al. 2024).

Graves’ disease and Hashimoto’s thyroiditis are autoimmune thyroid diseases with special therapeutic challenges on account of the underlying immune dysfunction (Banga and Schott 2018). A common feature is immune dysregulation, where, in Graves’ disease, autoantibodies stimulating the thyroid to produce an excessive amount of hormone and, in Hashimoto’s thyroiditis, the thyroid gland suffers autoimmune destruction (Barić et al. 2019). Treatment for Graves’ disease usually involves antithyroid medications, radioactive iodine, or surgical removal of the thyroid (Kolanu et al. 2024). The development of an optimal therapy is, however, complicated by certain side effects, including the possibility of post-treatment hypothyroidism and recurrence of hyperthyroidism following an initial successful treatment. Additional complications involve hormone replacement in Hashimoto’s thyroiditis, which usually involves the administration of levothyroxine. Such therapy may, however, require several adjustments in dosage to attain an optimal dosage level and must be followed for life (Trovato 2020). In both diseases, the treatment of the autoimmune component is cumbersome; therapies are largely symptomatic and directed at palliation rather than correction of the underlying immune dysfunction. New treatments targeting the modulation of the immune response are under investigation, but clinical application is still limited (Almahari et al. 2023).

The issue with all thyroid disorders, in general, is the lack of treatment options that can be more personalized. Although treatments are usually effective for managing thyroid function, individual genetic, environmental, or lifestyle factors that affect disease outcome and/or response to therapy are generally ignored. This is even the case with subclinical thyroid disorders in which there is a mild impairment in thyroid hormone levels without frank overt symptoms (Glenwright 2023). The question remains whether early intervention improves outcomes in such patients or leads to adverse effects due to unnecessary treatment. Most important, however, are the long-term consequences of thyroid hormone replacement and other therapies on bone health, cardiovascular health, and quality of life that are under-examined in this older population. Although increasing availability of genomic sequencing and biomarker research holds promise for more targeted therapies, much important research is needed about genetic predispositions of thyroid disorders and their impact on treatment response (Donoso 2018).

Despite these tremendous advances in diagnostic and therapeutic armamentarium, the conduct of thyroid disorders is still hampered by several limitations and challenges that include the difficulties of early and correct diagnosis-especially for autoimmune thyroid diseases-side effects of the current treatment modalities and imprecision in personalized care (Jacob et al. 2023). Each will be surmounted only as genetics, immunology, pathophysiology of thyroid diseases, diagnostic, minimally invasive treatments, and individually targeted therapeutic approaches improve in quality. It is only by this approach that clinicians may remain in the position of trying to afford their optimum available best quality service for improving life with high quality for patients with disorders of the thyroid gland (Knight et al. 2021).

### Key plants used for thyroid dysfunctions

The following sections and tables compile evidence from preclinical and clinical studies on natural interventions for thyroid disorders. A critical analysis of this evidence reveals a promising yet preliminary landscape. While numerous animal studies (*in vivo*) show significant effects on hormone levels and immune parameters, clinical trial data in humans remains limited for most interventions. The translation of these findings requires cautious interpretation and further rigorous human studies to establish efficacy, safety, and optimal dosing.

#### Hyperthyroidism

Hyperthyroidism is a disorder in which the thyroid gland in the neck generates an abnormally high level of thyroid hormones (T3 and T4), which are essential for controlling metabolism, energy, and development. When the thyroid is hyperactive, it accelerates several bodily activities, resulting in symptoms including tremors, anxiety, fast pulse, excessive perspiration, and weight loss. If left untreated, hyperthyroidism can lead to major issues such as heart disease, bone loss, and, in severe circumstances, a life-threatening thyroid storm. Graves’ illness, thyroiditis, or toxic nodular goiter are all potential causes. The disorder is normally diagnosed by blood tests to evaluate hormone levels, and treatment choices range from drugs and radioactive iodine to surgery, depending on severity (Lee and Pearce 2023).

As effective supplements or alternatives for conventional treatments for hyperthyroidism, a disorder marked by an overabundance of thyroid hormones, natural products have grown in favor. Although the major therapies are still medicine, radioactive iodine, and surgery (Gittoes and Franklyn 1998), some plants and herbs are essential for controlling thyroid function and reducing symptoms including anxiety, weight loss, and fast heartbeat (Khanum and Razack 2010). These natural products are widely known for their anti-inflammatory and antioxidant properties (Allegra 2019), which may complement traditional therapies by lowering inflammation, restoring hormone balance, and enhancing general health. Table 3 demonstrates plant-derived bioactive compounds/extracts known for their in vivo anti-hyperthyroidism activity.

**Table 3:** List of plants known for their anti-hyperthyroidism activity, depending on preclinical studies (*in vivo*)

No	Plant/bioactive compound	Extract type	Part used	Method	Results	References
	*Ocimum sanctum* Lamiaceae	Alcohol	Leaf	In vivo	Serum T4 content was considerably reduced by the plant extract when taken at a dose of 0.5 g kg^−1^ body weight for 15 days	(Panda and Kar 1998)
	*Rauwolfia serpentina* Apocynaceae	Alcohol	Root	In vivo	The plant extract (2.5 mg kg^−1^) was administered daily for 30 days and reduced thyroid hormone levels, suggesting its potential to regulate hyperthyroidism	(Panda and Kar 2000)
	*Convolvulus pluricaulis* Convulvulaceae	Alcohol	Root	In vivo	The administration of the plant extract resulted in a decrease in serum T3 concentration suggesting a potential regulatory effect of the plant extract on hyperthyroidism	(Panda and Kar 2001)
	*Aegle marmelos* Linn Rutaceae	Alcohol	Leaf	In vivo	Only the T3 concentration could be lowered by *A. marmelos* extract	(Kar et al. 2002)
	*Aloe vera* (L.) Liliaceae	Alcohol	Leaf	In vivo	*A. vera* inhibited serum levels of both T3 and T4	(Kar et al. 2002)
	*Emblica officinalis* Euphorbiaceae	Methanol	Fruit	In vivo	In comparison to propyl thiouracil (PTU), a common antithyroid medication, which decreased thyroid hormone levels by 59 and 40%, respectively, oral administration of the plant extract at a dose of 250 mg/kg/d (p.o.) for 30 days decreased T3 and T4 concentrations by 64 and 70%	(Panda and Kar 2003)
	Scopoletin	–	–	In vivo	Serum thyroid hormone levels were reduced in rats treated with levo-thyroxine when scopoletin (1.00 mg/kg, p.o.) was given daily for 7 days	(Panda and Kar 2006)
	*Annona squamosa* Annonaceae	Methanol extract	Seed	In vivo	It was clear from the data that administering L-T4 raised the levels of T3 and T4 in the blood. However, the benefits were reversed when 200 mg/kg of seed extract and an equivalent quantity of T4 were given together, as shown by a significant drop in the levels of both thyroid hormones	(Panda and Kar 2007)
	*Lagenaria siceraria* Cucurbitaceae	Ethanol	Peel	In vivo	The peel extracts inhibited serum thyroxine (T4) and triiodothyronine (T3) levels at a tolerable dosage of 100 mg/kg	(Dixit et al. 2008)
	*Bupleurum falcatum* Apiaceae	Water	Root	In vivo	When compared to LT4 control, the variations in blood T3, T4, and TSH concentrations caused by LT4 were considerably (*P* < 0.01) and dose-dependently normalized by 300, 150, and 75 mg/kg of BR extracts. Serum thyroid hormone levels were Likewise returned to normal by PTU 10 mg/kg, which was comparable to BR extracts 150 mg/kg	(Kim et al. 2012)
	Bugleweed Lamiaceae	Alcohol	Leaf	In vivo	T3 levels were significantly lowered by the extract (for over 24 h), most likely as a result of decreased peripheral T4 deiodination	(Al-Snai 2019)
	*Scutellaria baicalensis* Lamiaceae	Ethanol	Herb	In vivo	The ethanol extract of *Scutellaria baicalensis* has been proposed as an antithyroid medication since it suppresses both T3 and T4	(Kim and Lee 2019)
	Grape	Alcohol	Seed	In vivo	In animals with hyperthyroidism, oral GSE can considerably normalize the high levels of T3 and T4	(Albrahim and Robert 2020)
	Lemonbalm Lamiaceae	Methanol	Leaf	In vivo	Thyroid hormone levels significantly improved after receiving lemon balm extracts therapy. Additionally, it was successful in improving the thyroid gland’s histological appearance	(Mannaa et al. 2021)
	Cauliflower Brassicaceae	Water extract	Flower	In vivo	According to the current study’s findings, rabbits treated with cauliflower extract had significantly lower means of T3 and T4 hormone levels than those with hyperthyroidism (*P* < 0.05)	(Al-Jowari 2024)
	Korean red ginseng Araliaceae	Water extract	Root	In vivo	Korean red ginseng extract, as opposed to the propylthiouracil group, stopped reductions in body weight, thyroid gland weight, liver weight, blood glucose, and thyroid hormone levels in the levothyroxine-induced hyperthyroidism animal. The rise in blood aspartate aminotransferase, T3, and T4 levels following levothyroxine administration was also reduced. KRGE also improved liver and thyroid gland histopathology, which was not seen in the model of hypothyroidism caused by propylthiouracil	(Huang et al. 2024)
	*Prunella vulgaris* L Lamiaceae	Water extract	Whole plant	In vivo	TSH levels raised, whereas FT3 and FT4 amounts were decreased after treatment by the water extract	(Li et al. 2024)
	*Prunella vulgaris* L Lamiaceae	Water extract	Spica	In vivo	TSH levels raised, whereas FT3 and FT4 amounts were decreased after treatment by the water extract	(Li et al. 2024)

#### Hypothyroidism

Several herbs are identified with their positive role in the management of hypothyroidism. The administration of *Bacopa monnieri* (200 mg/kg B.wt.) has enhanced thyroid activity; it increases T4 levels as high as 41%. This herb is also known to improve memory, which has deteriorated during hypothyroidism (Kar et al. 2002). Research also indicates that extracts of *Withania somnifera* (1.4 g/kg B.wt.) and *Bauhinia purpurea* (2.5 mg/kg B.wt.) can stimulate thyroid activity in female mice. While *B. purpurea* enhances T3 and T4 hormones, *W. somnifera* primarily increases T4 levels (Panda and Kar 1999). In contrast, during the research with bipolar disorder patients, *W. somnifera* root surprisingly promoted the resolution of subclinical hypothyroidism (Gannon et al. 2014). Another study by Eshtiwi (2024) aimed to investigate the impact of a flavonoid extract from the seaweed *Fucus vesiculosus* L. on thyroid hormones in rats. Thirty-two mature rats were divided into four groups and administered varying doses of the seaweed extract over a month (50, 100, and 150 mg/cm^3^ of the extract/0.5 B.wt). The study assessed how the flavonoid extract from *F. vesiculosus* algae affected thyroid hormone levels in adult rats by measuring thyroid hormones, glutathione peroxidase, and peroxynitrite, along with liver enzymes in the blood serum. The findings revealed a notable decrease in TSH levels across all groups compared to the control group after a month of treatment. Additionally, T3 exhibited a significant increase in all groups compared to the control, as did T4. Glutathione peroxidase levels notably decreased in all groups compared to the control, while peroxynitrite levels also significantly decreased across all groups. Liver enzymes displayed a significant decrease in all groups compared to the control group (Eshtiwi 2024).

#### Thyroid nodules

As treatment methodologies for thyroid nodules are concerned, surgery and several non-surgical techniques such as radiofrequency ablation, alcohol injection, and high-intensity focused ultrasound Have been invented. Whereas about 95% of thyroid nodules are benign, the remaining ones mostly consist of well-treated thyroid Malignancies. Clinical interest in Having a new, non-invasive medical treatment to reduce thyroid nodule size does exist. A double-blind, placebo-controlled study for 3 months, carried out with 34 participants, found that a supplement containing Spirulina, Curcumin, and Boswellia extracts effectively diminished the size of benign thyroid nodules in euthyroid subjects. The selected patients with single thyroid nodules ranging from 2 to 5 cm were followed up over 12 weeks, which comprised 3 visits, 6 weeks apart from each other. During each visit, the size of the target thyroid nodule was measured, and plasma levels of thyroid-stimulating hormone, free thyroxin, and copper. The results obtained showed that the nodules became small in size (Stancioiu et al. 2019).

#### Goiter’s disease

Various natural products have shown their effect on Goiter’s disease; however, those studies are very few. For instance, Li et al. in 2023 confirmed the definite effect of three species of *Glycyrrhiza uralensis* in Haizao Yuhu decoction when treating goiter, and it was found that it was effective. It suppressed angiogenesis and cell proliferation in the goiter tissue due to its inhibition of the PI3K-Akt signaling pathway (Li et al. 2023). In contrast, experimental studies conducted about 60 years ago revealed that many plant parts can interact with thyroid function by inhibiting thyroid peroxidase—a key enzyme for the synthesis of thyroid hormones—or by suppressing the expression of thyroid-specific genes through competitive inhibition. Interference with the synthesis of thyroid hormones decreases the secretion of T3 and T4, which in turn increases the release of TSH. High levels of TSH promote the growth and activity of thyrocytes which may result in the enlargement of the thyroid gland known as goiter. Moudgal et al. (1958) for the first time reported the goitrogenic activity of some flavonoids (Moudgal et al. 1958). In a subsequent publication, Gaitan et al. demonstrated that endemic goiter, expressed in a West African population fed on a millet-rich diet, was due to the high content of glycosylated flavones in this food (Gaitan 1990).

#### Hashimoto’s thyroiditis

Saikosaponin-d is one of the active triterpenoids saponins isolated from the root of Bupleurum and has been widely used in Chinese medicine for its anti-inflammatory, immunoregulatory, and anti-tumoral activities. Thus, it has wide applications in a variety of autoimmune diseases with anti-inflammatory and immunoregulatory effects. The accumulated studies have indicated that saponin-d may improve lymphocytic infiltration in thyroid tissues and reduce serum TPOAb in mice with Hashimoto’s thyroiditis. Suggested mechanisms include maintenance of the balance between Th1/Th2 and Th17/Treg cells, induction of M2 macrophage polarization, and anti-inflammatory and tissue repair effects (Du et al. 2022).

Furthermore, total glucosides of *Paeonia lactiflora* (TGPL) are key bioactive components extracted from *Paeonia lactiflora*, known for their anti-inflammatory, immunomodulatory, and hepatoprotective activities. TGPL has been suggested for combating autoimmune diseases like systemic lupus erythematosus, psoriasis, and HT. In a rat model of autoimmune thyroiditis, TGPL significantly lowered serum TPOAb, TGAb, and TNF-α levels while boosting IL-10 levels and ameliorating thyroid follicle damage. The potential impact of TGPL on HT could be linked to enhanced intestinal flora diversity and reduced mucosal barrier damage, although direct connections between microbiota composition and thyroid autoimmunity require further investigation (Mu et al. 2021). Also, ginsenosides, the active components of ginseng, exhibit protective effects against inflammation, oxidative stress, and apoptosis, making them promise to treat various conditions. In rat models of autoimmune thyroiditis, ginsenosides have demonstrated efficacy in reducing thyroid autoantibody titers by modulating cytokine expression and promoting a balanced Th1/Th2 ratio (Chen et al. 2016). Moreover, *Astragalus membranaceus*, known as Huangqi in Chinese medicine, has been extensively used to address HT. Studies highlight its anti-inflammatory effects against inflammation, thyrocyte damage, and apoptosis. Clinical trials have demonstrated that Huangqi Capsule, in combination with an iodine-restricted diet, can significantly decrease TPOAb and TGAb titers compared to diet alone in HT patients, providing valuable human data. The selenium-rich components of *A. membranaceus* may enhance the gastrointestinal barrier, potentially increasing selenium bioavailability (Zhang et al. 2016). As well, *Dioscorea nipponica makino*, a medicinal herb used in traditional Chinese medicine, is another notable adjunct therapy for thyroid conditions like HT. *D. nipponica makino* granules, derived from its extract, have shown promise in reducing thyroid autoantibody levels and rebalancing the immune system in patients with thyroid diseases. Clinical studies suggest that these granules, when combined with L-T4, can significantly lower TPOAb and TGAb titers, Th17 cells, and relevant cytokines compared to L-T4 treatment alone, while also enhancing the Treg/Th17 ratio, representing another intervention with supporting clinical evidence (Cao et al. 2016).

#### Graves’ disease

Cepharanthine is an alkaloid naturally extracted from the plant *Stephania cepharantha* Hayata. It has been reported to suppress the T cell activation induced by Tg peptides in immunized non-obese diabetic mice with the DRb1-Arg74 variant of HLA-DR known to be associated with risk of autoimmune thyroid disease, in response to human Tg. Stimulated by these encouraging results, a novel dual-action polymeric system is proposed for the potential treatment of autoimmune thyroid disease. This system is designed for the co-delivery of cepharanthine and selenium. Cepharanthine would target and inhibit T cell activation, addressing the underlying autoimmune response, while selenium would function to lower TPOAb levels and provide antioxidant support. This combinational approach aims to synergistically modulate the immune system and reduce thyroid cell damage, though it remains a theoretical proposal requiring extensive preclinical validation. Recent studies have observed that cepharanthine is capable of inhibiting the interaction between TSHR.132 (TSH receptor peptide) and HLA-DRβ1-Arg74, thus blocking T cell activation and cytokine response in a Graves’ disease-relevant humanized mouse model expressing HLA-DR3 (Li et al. 2020). Table 4 demonstrates plant-derived bioactive compounds/extracts known for their preclinical and clinical anti hypothyroidism, thyroid nodules, and autoimmune disorders of thyroid.

**Table 4:** Preclinical and clinical evidence of medicinal plants against hypothyroidism, thyroid nodules, and autoimmune disorders of thyroid

Name of plant/bioactive compound	Type of extract/part used	Methods	Results	References
**Hypothyroidism**				
***Fucus vesiculosus* L**	Flavonoid extract of *Fucus vesiculosus* L. seaweed	In vivo	Increase in T3 and T4 levels	(Eshtiwi 2024)
***Bacopa monnieri***	Leaf extracts	In vivo	Increase in T_4_ concentration. Decrease in hepatic LPO. Increase in SOD and CAT activities	(Kar et al. 2002)
**Goiter**				
**Haizao Yuhu decoction of *Glycyrrhiza uralensis***	Whole plant extract	In vivo	Decrease in absolute/relative weights of thyroid tissue. Improvement in pathological structure and thyroid function	(Li et al. 2023)
**Thyroid nodules**				
**Spirulina, Curcumin, Boswellia**	Supplement of combined extracts	Clinical trial	Decrease in the size of benign thyroid nodules	(Stancioiu et al. 2019)
**Hashimoto’s thyroiditis**				
**Saikosaponin-d**	Bupleurum extract	In vivo	Decrease in TPOAb, decrease in IFN-γ and IL-17	(Du et al. 2022)
**Total glucosides**	*Paeonia lactiflora*	In vivo	Decrease in TPOAb and TGAb. Decrease in TNF-α. Increase in IL-10	(Mu et al. 2021)
**Ginsenosides**	Ginseng	In vivo	Decrease in TPOAb and TGAb. Decrease in IFN-γ and IL-2. Increase in IL-4	(Chen et al. 2016)
***Astragalus membranaceus***	Dried roots	Clinical trial	Decrease TPOAb and TGAb	(Zhang et al. 2016)
***Dioscorea nipponica makino***	Dried tubers	Clinical trial	Decrease TPOAb and TGAb. Increase in FT3 and FT4. Decrease in TSH. Decrease in TCM syndrome score. Increase in total effective rate	(Cao et al. 2016)
**Graves’ disease**				
**Cepharanthine**	*Stephania cepharantha Hayata*	In vivo	Inhibition in T cell activation triggered by Tg peptides in non-obese diabetic mice that express DRb1-Arg74, a variant of HLA-DR	(Li et al. 2020)
***Withania somnifera***	Root extract	In vivo	Increase in T4 concentration. Increase in G-6-Pase activity and decrease in LPO. Increase in the activity of antioxidant enzyme(s)	(Panda and Kar 1999)
***Bauhinia purpurea***	Bark extract	In vivo	Increase in T_3_ and T_4_. Increase in G-6-Pase activity. Decrease in LPO	(Panda and Kar 1999)

#### Thyroid cancer

Thyroid carcinoma is the most prevalent endocrine malignancy, with steadily increasing incidence, partly due to enhanced diagnostic techniques detecting smaller tumors, showing higher rates in women than in men (Prete et al. 2020). It encompasses several malignancies, primarily differentiated by cell type and behavior. The main types include the predominant papillary thyroid carcinoma (PTC) that accounts for 85% of the reported cases characterized by its slow growth and excellent prognosis, the follicular thyroid carcinoma (FTC) that represents approximately 10–15% of the cases and is more likely to metastasize via bloodstream to other organs, the less common medullary thyroid carcinoma (MTC) which arises from parafollicular C-cells and is often linked to genetic syndromes, and the rare but aggressive anaplastic thyroid carcinoma (ATC) with poor prognosis due to rapid progression and resistance to treatments (Haddad, Elisei et al., Schmidbauer et al. 2017; Coca-Pelaz et al. 2020; Prete et al. 2020).

Natural products derived from plants have shown promising results in thyroid cancer treatment. Phytochemicals such as flavonoids, alkaloids, and polyphenols have demonstrated the ability to modulate signaling pathways involved in cancer cell proliferation, apoptosis, and metastasis. Curcumin, for instance, has been reported to inhibit tumor growth and induce apoptosis in thyroid carcinoma cell lines. Resveratrol and quercetin also exhibit anti-proliferative effects, providing potential complementary approaches to traditional therapies like surgery and radioactive iodine (Sharifi-Rad et al. 2020; Kaczmarzyk et al. 2024).

Despite advancements in conventional treatments, including targeted therapies for aggressive types like ATC and MTC, integrating plant-based compounds remains a promising area of research. These natural products not only have lower toxicity profiles but also enhance the efficacy of existing therapies. Continued studies are necessary to elucidate their mechanisms and optimize their application in personalized medicine.

In this section, 30 articles were reviewed to highlight 50 plant-derived bioactive compounds/extracts as listed in Table 5 that are reported to have anticancer activity against thyroid carcinoma, showing their phytochemical class, botanical source, method of bioactivity assessment, and their reported results and mechanism of action by which they exerted their activity.

**Table 5:** Preclinical evidence of plant-derived bioactive compounds/extracts reported to have anticancer activity against thyroid carcinoma

No	Bioactive compound/extract	Source	Method	Results	Reference
***Diarylheptanoid***					
**1**	Curcumin	Turmeric (*Curcuma longa*)	In vitro and in vivo against PTC, FTC, ATC, MTC	Inhibition of PI3K/Akt signaling pathway, induction of apoptosis, suppression of cell proliferation and migration	(Perna et al. 2018, Sharifi-Rad et al. 2020; Bulotta et al. 2021)
***Stilbenoid***					
**2**	Resveratrol	Grapes	In vitro and in vivo against PTC, FTC, ATC, MTC	Induction of apoptosis, cell cycle arrest, inhibition of angiogenesis, modulation of various signaling pathways (e.g., NF-κB, PI3K/Akt)	(Shin et al. 2019; Sharifi-Rad et al. 2020)
**3**	Combretastatin	*Combretum leprosum*	In vitro and in vivo studies against MTC, PTC, ATC	Delayed the growth of MTC tumor	(Lichota and Gwozdzinski 2018; Sharifi-Rad et al. 2020; Bulotta et al. 2021)
***Flavonoids and**** their**** derivatives***					
**4**	Quercetin	Apples, onions, berries, tea	In vitro and in vivo against PTC, FTC, ATC	Induction of apoptosis, cell cycle arrest, inhibition of angiogenesis	(Sharifi-Rad et al. 2020; Du and Shen 2024)
**5**	Apigenin	Parsley, celery, chamomile tea	In vitro and in vivo against PTC, ATC	Induction of apoptosis, cell cycle arrest, inhibition of angiogenesis, increase iodide influx rate	
**6**	Myricetin	Berries, tea, nuts	In vitro and in vivo against PTC, FTC, and ATC	Induction of apoptosis, cell cycle arrest, inhibition of angiogenesis, reduced cell proliferation, by 70%. The mechanism involved an increase in the activation of caspase-dependent apoptosis and altered the mitochondrial membrane potential	(Ha et al. 2017; Jo et al. 2017)
**7**	Genistein	Soybeans, legumes	In vitro and in vivo against PTC and MTC	Inhibition of cell proliferation, induction of apoptosis	(Sharifi-Rad et al. 2020; Du and Shen 2024)
**8**	Daidzein	Legumes	In vitro and in vivo against MTC, ATC	Inhibit cell proliferation, induce apoptosis	
**9**	Xanthohumol	*Humulus lupulus* (Hops)	In vitro against PTC	At 10 μM, stops or slows down cell division, preserving the viability of the cells. At 100 μM, a decrease of cell viability by induction of apoptosis. Induced DNA fragmentation and promoted cell cycle arrest	(Carvalho et al. 2018)
**10**	Baicalein	Roots of *Scutellaria baicalensis* Georgi and *Scutellaria lateriflora* L	In vitro against ATC	Induction of apoptosis, reduced proliferation	(Sharifi-Rad et al. 2020; Du and Shen 2024)
**11**	Chrysin	*Oroxylum indicum* (L.) Kurz, *Passiflora caerulea* L., *Passiflora edulis* Sims, *Matricaria chamomilla* L., honey, propolis	In vitro against ATC	Induction of apoptosis, reduced proliferation	
**12**	Nobiletin	*Citrus* peel	In vitro against PTC and ATC	Suppress proliferation and migration of PTC cells, reduce cell viability of ATC at 100 μM comparable to conventional drugs with less toxicity	(Du and Shen 2024)
**13**	Hesperitin	*Citrus* fruits	In vitro against PTC, ATC	Activates caspase-mediated apoptosis and reduces oxidative stress	(Kaczmarzyk et al. 2024)
**14**	Luteolin	Celery, green pepper, broccoli, carrots, and olive oil	In vitro against PTC and FTC	Suppress tumor progression	(Liu et al. 2017)
**15**	Naringenin	Citrus fruits	In vitro against PTC	Induce cell apoptosis	(Kaczmarzyk et al. 2024)
**16**	Deguelin	Legumes	In vitro against ATC	Suppress spheroid formation and cellular motility, induce apoptosis	(Sharifi-Rad et al. 2020, Li et al. 2022)
***Gallic acid derivatives***					
**17**	EGCG (Epigallocatechin gallate)	Green tea	In vitro and in vivo against PTC, FTC	Induction of apoptosis, cell cycle arrest, inhibition of angiogenesis	(Sharifi-Rad et al. 2020; Bulotta et al. 2021)
**18**	Punicalagin	Pomegranate	In vitro and in vivo against PTC	Induce autophagic cell death, cell cycle arrest	(Bulotta et al. 2021)
**19**	Ellagic acid	Pomegranate, berries	In vitro against ATC	Induce apoptosis, inhibit cell proliferation, migration, and invasion	(Bulotta et al. 2021; Meng et al. 2023)
***Steroids***					
**20**	Withanolides	*Withania somnifera* (L.) Dunal	In vitro and in vivo against MTC	Significant tumor regression, growth delay, and calcitonin level decrement, inhibiting the phosphorylation of RET, ERK, and AKT kinases, selective prevention of RET phosphorylation, AKT/mTOR pathway activation, and protein synthesis capacity of MTC cells	(Sharifi-Rad et al. 2020)
***Isothiocyanate***					
**21**	Sulforaphane	Cruciferous vegetables (broccoli, cauliflower, cabbage)	In vitro and in vivo against PTC	Induction of phase II detoxification enzymes, induce apoptosis, inhibition of cell proliferation and migration, modulate oxidative stress	(Wang et al. 2015)
***Alkaloids***					
**22**	Aloperine	*Sophora alopecuroides* L	In vitro against ATC, PTC, FTC	Induction of apoptosis, cell growth inhibition	(Sharifi-Rad et al. 2020; Di Dalmazi et al. 2024)
**23**	Indirubin	*Indigofera tinctoria* L	In vitro against ATC, FTC, PTC, MTC	Induction of apoptosis, inhibit tumor growth	
**24**	Evodiamine	*Tetradium ruticarpum* (A.Juss.) T.G.Hartley, unripe fruits of *Evodia rutaecarpa* (Juss.) Benth	In vitro and in vivo against ATC	Induction of apoptosis, inhibit tumor growth, induce autophagy, block metastasis	
**25**	Capsaicin	Genus* Capsicum*	In vitro against PTC, ATC	Preventing thyroid cancer cell metastasis	
**26**	Piperine	*Piper nigrum* L. (black pepper) and *Piper longum* L. (long pepper)	In vitro against PTC	Inhibit cancer cell proliferation	
**27**	Berberine	Barberry (*Berberis* spp.)	In vitro against PTC, ATC	Induce cell apoptosis and autophagy, induced significant mitochondrial apoptosis, G0/G1 cell cycle arrest and inhibit migration	(Park et al. 2012; Li et al. 2017)
**28**	Camptothecin	Stem wood of the Chinese tree, *Camptotheca acuminata*	In vitro and in vivo against ATC	Decreased tumor growth, metastatization, and tumor microvessel density	(Di Dalmazi et al. 2024)
**29**	Harmine	Seeds of *Peganum harmala*	In vitro and in vivo against PTC	Decreased tumor growth	
**30**	Matrine	Genus *Sophora*	In vitro and in vivo against PTC, FTC	Decreased tumor growth	
**31**	Piperlongumine	*Piper longum* L	In vitro and in vivo against PTC, ATC, FTC	Decreased tumor growth, inhibited cell proliferation and colony formation, promoted cell cycle arrest and cellular apoptosis via caspase-dependent pathway	
**32**	Colchicine	*Colchicum autumnale*	In vitro against PTC	Inhibited the tumor cell growth, induced apoptosis	(Di Dalmazi et al. 2024)
**33**	Cyclopamine	*Veratrum californicum*	In vitro against FTC, ATC	Inhibited tumor cell growth, induced apoptosis, decreased cell migration	(Parascandolo et al. 2018)
**34**	Ellipticine	*Ochrosia elliptica* and other Apocynaceae plants	In vitro and in vivo against MTC	Inhibit tumor growth, suppress proliferation	(Di Dalmazi et al. 2024)
**35**	Sanguinarine	*Sanguinaria canadensis*,* Argemone mexicana*	In vitro against PTC	Inhibited the tumor cell growth, induced apoptosis, sensitized PTC cells to chemotherapeutic drug cisplatin	
***Terpenes and its derivatives***					
**36**	Ursolic acid	Abundant in apple wax, basil, rosemary, oregano, thyme…etc	In vitro against PTC, MTC	Inhibit proliferation, migration and invasion of tumor	(Cao and He 2022; Kaczmarzyk et al. 2024)
**37**	Oleuropein	*Oleaceae* plant species	In vitro against PTC	Reduced cell proliferation	(Bulotta et al. 2021)
***Lectins***					
**38**	*Maackia amurensis*	F. Fabaceae	In vitro against ATC, PTC	Inhibition of the proliferative, invasive and malignant character of ATC + necrotic cell death without any toxicity for normal thyroid epithelial cells, decreased the mobility in PTC	(Kaptan et al. 2018)
**39**	*Sambucus nigra*	F. Adoxaceae	In vitro against PTC	Decreased the mobility in PTC	
***Miscellaneous compounds***					
**40**	Indole-3-carbinol (I3C) and its dimer	Produced upon hydrolysis/chewing of Cruciferous vegetables	In vitro against PTC	Inhibiting cell survival of carcinoma cells mediated by G1 arrest followed by induction of apoptosis	(Fares 2014)
**41**	Shikonin	Root extract of *genera Arnebia*, *Lithospermum*, and* Onosma*	In vitro against MTC	Induce apoptosis, decreased proliferation, anti-invasive effect	(Sharifi-Rad et al. 2020)
**42**	Thymoquinone	Black seed (*Nigella sativa*)	In vitro and in vivo against PTC, ATC	Induce apoptosis	(Ozturk et al. 2018)
**43**	Hydroxytyrosol	*Olea europaea*	In vitro against PTC	Reduce cancer cell viability, promoted apoptotic cell death	(Bulotta et al. 2021)
***Extracts***					
**44**	*Stemona tuberosa* Lour	Crude root extract	In vitro against MTC	Induce apoptosis, LC_50_ was 50 µg/ml	(Rinner et al. 2004)
**45**	*Aglaia tenuicaulis* Hiern	Crude extract	In vitro against MTC	Antiproliferative effects, LC_50_ was 30 µg/ml	
**46**	*Prunella vulgaris*	Crude flower extract	In vitro and in vivo against PTC	Inhibit the proliferation and migration of cancer cells both in vitro andin vivo	(Zheng et al. 2021)
**47**	*Punica granatum*	Pomegranate peel crude extract	In vitro and in vivo against PTC	Suppressed proliferation, induced cancer cell apoptosis via a mitochondria-mediated apoptotic pathway, impaired cancer cell migration and significantly inhibited tumor growth	(Li et al. 2016)
**48**	*Vitis vinifera*	Grape stem defatted extract of Voidomato and Muscat varieties	In vitro against thyroid cancer cells	Inhibited cell proliferation, with IC_50_ value 159–314 µg/ml	(Sahpazidou et al. 2014)
**49**	*Pulsatilla koreana*	Root extract	In vitro and in vivo against ATC	Induction of apoptosis, anti-angiogenesis	(Park et al. 2013)
**50**	*Clitoria ternatea*	Crude extract	In vitro against MTC	Induced apoptosis, IC_50_ = 55 µg/ml	(ALshamrani et al. 2022)

## Conclusions and future prospects

Despite the potential of medicinal plants in treating thyroid disease, there is a limited amount of research addressing their efficacy. The specific mechanisms by which these plants may protect tissues from drug toxicity, exert antioxidant effects, regulate immune function, and control thyroxine levels remain largely unexplored. Consequently, it is crucial to design systematic experimental approaches to investigate how natural products influence thyroid disease and the associated physiological mechanisms. In summary, this review has identified several natural products—such as *Withania somnifera*, *Ocimum sanctum*, curcumin, and *Astragalus membranaceus*—that have demonstrated significant effects on thyroid function or pathology in preclinical models, with some like *Astragalus* and *Dioscorea nipponica* showing promise in preliminary clinical studies. These findings underscore the potential of natural adjuvants in thyroid therapy, particularly due to their multi-target effects (e.g., anti-inflammatory, immunomodulatory, and antioxidant) which align well with the complex pathophysiology of thyroid disorders. The therapeutic effects of medicinal plants could significantly enhance clinical management of thyroid disorders while minimizing drug-induced side effects and improving overall treatment outcomes. Therefore, a thorough investigation into the molecular mechanisms of these herbal remedies is vital for advancing the treatment and prognosis of thyroid disease. Future studies should prioritize (1) isolating and characterizing the active compounds within these botanicals, (2) elucidating their precise molecular targets (e.g., thyroid peroxidase, sodium-iodide symporter, cytokine signaling pathways), and (3) conducting well-designed controlled clinical trials to rigorously evaluate their safety and efficacy in human patients. Special attention should be paid to standardizing extracts, determining optimal dosing, and investigating potential herb-drug interactions. Furthermore, exploring novel delivery systems, such as the proposed cepharanthine-selenium polymer, could enhance bioavailability and therapeutic efficacy. By elucidating these pathways, we can better understand how these natural products can be integrated into conventional therapies, potentially leading to more effective and safer treatment options for patients with thyroid dysfunctions.

## Data Availability

All source data for this work (or generated in this study) are available upon reasonable request.

## Abbreviations

AITD: Autoimmune thyroid disease
CAT: Catalase
FNA: A fine needle aspiration
G-6-Pase: Glucose-6-phosphatase
GD: Graves’ disease
GM: Gut microbiota
HT: Hashimoto’s thyroiditis
IgG: Immunoglobulin G
L-T4: Levothyroxine
NIH: National Institutes of Health
PTU: Propylthiouracil
RAI: Radioactive iodine therapy
SOD: Superoxide dismutase
T3: Triiodothyronine
T4: Thyroxine
TCM: Traditional Chinese medicine
TGAb: Thyroglobulin antibodies
Th1/Th2: T-helper 1/T-helper 2
Th17/Treg: T-helper 17/regulatory T cells
TPOAb: Thyroid peroxidase antibodies
TSH: Thyroid-stimulating hormone
TSI: Thyroid stimulating immunoglobulin
